# A review of physical and engineering factors potentially affecting shear wave elastography

**DOI:** 10.1007/s10396-021-01127-w

**Published:** 2021-08-28

**Authors:** Naotaka Nitta, Makoto Yamakawa, Hiroyuki Hachiya, Tsuyoshi Shiina

**Affiliations:** 1 Health and Medical Research Institute, National Institute of Advanced Industrial Science and Technology (AIST), 1-2-1 Namiki, Tsukuba, Ibaraki 305-8564 Japan; 2 Graduate School of Medicine, Kyoto University, Kyoto, 606-8507 Japan; 3 School of Engineering, Tokyo Institute of Technology, Meguro, Tokyo, 152-8552 Japan

**Keywords:** Shear wave elastography, Shear wave speed, Interpretation, Physical factors, Engineering factors

## Abstract

It has been recognized that tissue stiffness provides useful diagnostic information, as with palpation as a screening for diseases such as cancer. In recent years, shear wave elastography (SWE), a technique for evaluating and imaging tissue elasticity quantitatively and objectively in diagnostic imaging, has been put into practical use, and the amount of clinical knowledge about SWE has increased. In addition, some guidelines and review papers regarding technology and clinical applications have been published, and the status as a diagnostic technology is in the process of being established. However, there are still unclear points about the interpretation of shear wave speed (SWS) and converted elastic modulus in SWE. To clarify these, it is important to investigate the factors that affect the SWS and elastic modulus. Therefore, physical and engineering factors that potentially affect the SWS and elastic modulus are discussed in this review paper, based on the principles of SWE and a literature review. The physical factors include the propagation properties of shear waves, mechanical properties (viscoelasticity, nonlinearity, and anisotropy), and size and shape of target tissues. The engineering factors include the region of interest depth and signal processing. The aim of this review paper is not to provide an answer to the interpretation of SWS. It is to provide information for readers to formulate and verify the hypothesis for the interpretation. Therefore, methods to verify the hypothesis for the interpretation are also reviewed. Finally, studies on the safety of SWE are discussed.

## Introduction

It has been recognized that tissue stiffness provides useful diagnostic information, as with palpation as a screening for diseases such as cancer. In recent years, various elastography techniques for evaluating and imaging tissue elasticity quantitatively and objectively in diagnostic imaging have been put into practical use, and the amount of clinical knowledge has increased [1–3]. In addition, some diagnostic guidelines have been issued [4], many review papers on technology and clinical applications have been published [5–10], and the status as a diagnostic technology is in the process of being established.

Ultrasound elastography techniques currently available in clinical practice include strain imaging [11], which measures and images the strain generated inside tissue by manually applying static compressive forces; acoustic radiation force impulse (ARFI) imaging [12], which images the displacement distribution generated by the acoustic radiation force (ARF); shear wave elastography (SWE) [13–16], which measures the shear wave speed (SWS) generated by the ARF; and transient elastography (TE) [17–20], which measures the SWS generated by applying external vibrations using an actuator. In the former two technologies (strain imaging and ARFI imaging), if the stress or force in the tissue is constant, the strain or displacement is small in the hard tissue and large in the soft tissue. These provide relative evaluations, and the stiffness cannot be evaluated as an absolute value. In the latter two technologies (SWE and TE), on the other hand, if the density in the tissue is constant, the SWS is higher in the hard tissue and lower in the soft tissue. In addition, under the assumption that the tissue is almost incompressible, it is easy to convert to the elastic modulus such as Young’s modulus, and it is possible to absolutely evaluate the stiffness of tissue. Therefore, expectations for the clinical significance of SWE are increasing. However, there are still unclear points about the interpretation of the measured SWS and converted elastic modulus. To clarify these unclear points, it is important to investigate the factors that affect the SWS and elastic modulus, understand the mechanism related to the behavior of shear wave propagation, and formulate and verify the hypothesis.

Therefore, physical and engineering factors that potentially affect the SWS and the converted elastic modulus are discussed in this review paper, based on the principles of SWE and a literature review. The aim of this review paper is not to provide an answer to the interpretation of SWS. It is to provide information for readers to formulate and verify the hypothesis for the interpretation.

The remainder is organized as follows. First, an overview of clinical applications of SWE to date is given. Next, the principle of SWE is described, and the physical and engineering factors that potentially affect the SWS and the converted elastic modulus are reviewed based on the literature. After that, some methods for verifying the hypothesis with respect to the interpretation of SWS and elastic modulus are described. Finally, some studies on the safety of SWE are discussed as supplemental information.

## Overview of clinical application of SWE

In principle, although SWE may be applied to any tissue capable of generating effective shear waves, the targets for the application of SWE at present are mainly breast and liver tissue. Initial application to the breast was reported by Tanter et al. [21]. In addition, there are many reports on the application of SWE to the liver. Liver biopsy has traditionally been performed as a reliable method [22], but with the advent of TE, SWE, and magnetic resonance elastography (MRE), expectations are increasing as a noninvasive alternative to liver biopsy [23]. FibroScan^®^ is expected to be a noninvasive assessment of liver fibrosis [24], but it has also been reported that it may fail in obese patients [25]. In the application of SWE to the liver, its feasibility [26] and capability to classify the stages of fibrosis in patients with nonalcoholic fatty liver disease (NAFLD) [27] have been investigated. In addition, the reliability of diagnosis according to the liver site (right and left) has been investigated [28], and the possibility of detecting pancreatic cancer in animal experiments has been reported [29]. In addition, as examples of application to organs other than the breast and liver, SWS of the placenta after delivery [30] and SWS for muscles (described later) have been reported. The reproducibility of SWE may vary depending on the part of the liver [31] and seems to be dependent on the operator. In particular, it has been reported that the reliability and reproducibility of skilled or trained operators are high [32, 33]. Also, there are reports that SWE and FibroScan^®^ show a good correlation [34, 35]. On the other hand, in terms of detectability of lesions, depending on various factors such as the patient’s disease status and measurement position, some results show that SWE is superior to FibroScan^®^, while other results show that FibroScan^®^ is superior to SWE [36–39].

While SWE allows quantitative evaluation, it is important to consider various factors regarding the reliability, reproducibility, and interpretation of the SWS. The following sections provide an overview of physical and engineering factors that potentially affect SWE, which have been reported to date, based on the principles of SWE.

## The principles of SWE

The principles of SWE are as follows. First, a focused pulse wave (push pulse) with a longer duration (pulse width) than that used in conventional ultrasonic diagnostic equipment is transmitted near the region of interest (ROI), and then the shear wave is generated by the ARF. Next, the SWS, i.e., the propagation speed of the generated shear wave in the lateral direction perpendicular to the push pulse direction, is measured, and the SWS is converted into an elastic modulus such as Young’s modulus.

### Shear wave generation

When the push pulse is transmitted, the ARF ($F$) is applied at the focal point along the progressive direction of the push pulse, as shown in Eq. (1).

1 $$F=\frac{2\alpha I}{{c}_{\mathrm{l}}}.$$

**Fig. 1:**
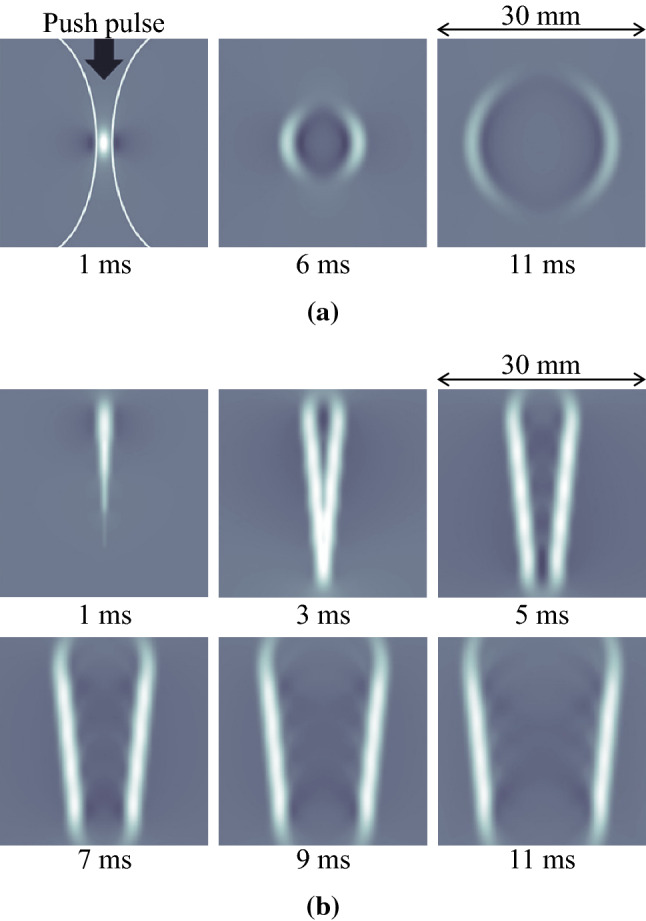
Propagation of shear waves generated by push pulse transmission. **a** Propagation of a shear wave generated by a push pulse with a single focus. **b** Propagation of a plane shear wave generated by continuously transmitting multiple push pulses with different focal points

Here, $\alpha$ is the absorption coefficient, $I$ is the time average intensity of the push pulse, and ${c}_{\mathrm{l}}$ is the longitudinal wave speed. The duration of the push pulse is usually several hundred microseconds, which is sufficiently longer and has higher energy than the conventional image pulse. Due to the generated ARF, the displacement occurs in the progressive direction of the push pulse. Then, when the transmission of the push pulse is completed, the displaced tissue returns to the original position. The shear wave is generated by this series of vertical tissue displacement and propagates in a spherical wave shape spreading from the focal point, as shown in Fig. 1a. In general, it is considered that the displacement generated by the push pulse in the progressive direction increases as the time average intensity of the push pulse increases, and the amplitude of the shear wave also increases accordingly.

In the push pulse transmission with a single focal point, the region in which the SWS distribution can be obtained may be narrow due to the large attenuation of the shear wave. Therefore, the push pulse transmission is improved to widen the measurement region of the SWS. Bercoff et al. have generated the plane shear wave with a large amplitude, as shown in Fig. 1b, by setting multiple focal points on the ultrasonic beam axis and continuously transmitting push pulses to each focal point [15]. Song et al. have shown that a wide region of SWS distributions can be obtained by simultaneously transmitting multiple unfocused push pulses in a comb shape (comb push) [40]. A method using a spatially modulated ARF has also been proposed [41].

### Measurement of SWS

**Fig. 2:**
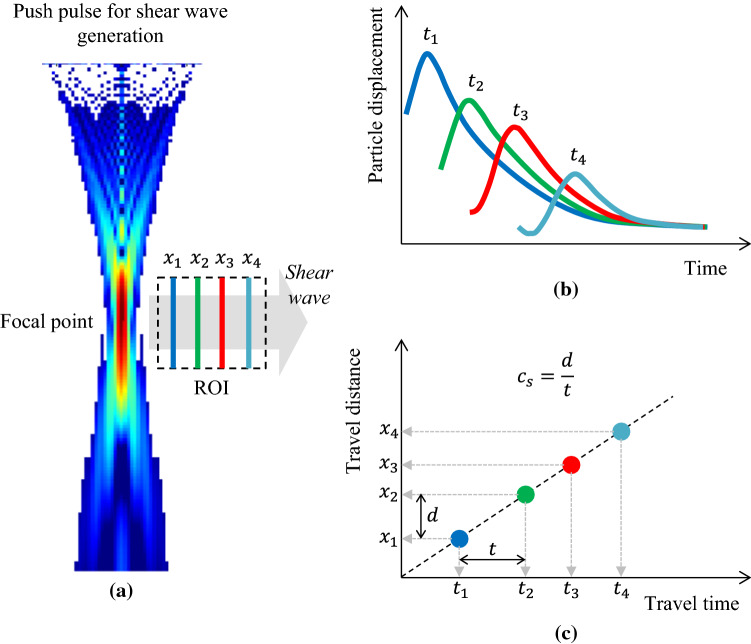
Outline of propagation speed measurement of shear waves generated by push pulse transmission. **a** Positions of scan lines (${x}_{1}$, ${x}_{2}$, ${x}_{3}$, ${x}_{4}$) in measuring the particle displacement of shear waves. **b** Transition of particle displacement waveforms and its peak time transition (travel time) (${t}_{1}$, ${t}_{2}$, ${t}_{3}$, ${t}_{4}$) obtained at each scan line. **c** Relationship between the propagation distance of a shear wave (travel distance) and travel time. The slope of this straight line corresponds to the average value of the SWS in the ROI

The methods for measuring the SWS in tissue include point SWS measurement (or point SWE), which measures the average value of the SWS in an ROI, and SWS imaging (or 2D SWE), which images the SWS distribution in the ROI [1]. An outline is shown in Fig. 2. Since the direction of the particle displacement of a shear wave is basically included in the axial direction of the ultrasonic beam for tracking, the shear wave can be tracked by the same concept as conventional ultrasound Doppler blood flow measurement (phase shift detection). The SWS in soft tissue is approximately 1–10 m/s. When considering the restrictions on the pulse repetition frequency (PRF) of the tracking pulse and frame rate in 2D SWE, the region (ROI) of the SWS measured by the conventional phase shift detection (line-by-line transmission) becomes narrow. Therefore, a high-speed imaging method (ultrafast imaging method) that transmits the plane waves and visualizes the shear wave propagation has been proposed [15, 42]. In imaging using plane wave transmission, unlike conventional line-by-line scanning, an unfocused plane wave formed by driving all elements on the ultrasound probe is transmitted, the beamforming is performed in only the receiving process, and the image is reconstructed. Therefore, although the image quality deteriorates compared to conventional line-by-line scanning, theoretically, the frame rate becomes equivalent to the PRF, and the particle displacement distribution in the shear wave propagation can be measured over a wide region.

As described above, since the distribution of the particle displacement in the shear wave propagation can be measured by the same concept as the conventional phase shift detection, many potential methods for tracking the movement have been proposed [43–48]. In the case of typical phase shift detection, two consecutive tracking pulses at a certain PRF are transmitted on the same scan line during the shear wave propagation, and the corresponding two received echoes are obtained. Assuming that the in-phased and quadrature signals of the first received echo is $\left({I}_{1},{Q}_{1}\right)$, and those of the second received echo are $\left({I}_{2},{Q}_{2}\right)$, the phase shift $\Delta \phi$ between the two signals is calculated by Eq. (2).

2 $$\Delta \phi ={\mathrm{tan}}^{-1}\left(\frac{{I}_{1}{Q}_{2}-{Q}_{1}{I}_{2}}{{I}_{1}{I}_{2}+{Q}_{1}{Q}_{2}}\right).$$

The phase shift is converted to the particle displacement. The waveforms of the particle displacement are obtained on the scan lines of two points, $x={x}_{1}$ and $x={x}_{2}\left(={x}_{1}+d\right)$, which are separated by a distance $d$ in the lateral direction ($x$). When the time $t$ that the waveforms of particle displacement propagate from ${x}_{1}$ to ${x}_{2}$ is obtained using a cross correlation function, the SWS ${c}_{s}$ can be obtained by Eq. (3).

3 $${c}_{s}=\frac{d}{t}.$$

However, it may be difficult to stably measure the SWS using this time of flight (TOF) method due to the noise contained in the measurement of shear wave propagation. Therefore, robust estimations have been proposed to improve accuracy and precision [49, 50]. In addition to the TOF method, a method of estimating the wavelength of shear waves has also been proposed [51].

Reflection and refraction of the shear wave occurs at the boundary in tissues where the acoustic impedance is different, and the estimation of the SWS may fail due to the interference of the progressive and reflected shear waves. When the amplitude of the shear wave becomes small due to the interference, the time shift of particle displacement waveforms may become difficult to identify. Therefore, a directional filter that separates the progressive and reflected components from the interference of the shear wave has been proposed [52–56]. When the directional filter is applied as a preprocessing for calculating the SWS, only the progressive component of the shear wave is extracted from the interference wave, thus stabilizing measurement of the SWS.

### Calculation of elastic modulus

Assuming that the tissue is an isotropic linear elastic body, the SWS ${c}_{\mathrm{s}}$ is expressed by Eq. (4).

4 $${c}_{\mathrm{s}}=\sqrt{\frac{G}{\rho }}.$$

Here, $G$ is the shear modulus, and $\rho$ is the density. Further, the longitudinal elastic modulus (or Young’s modulus) $E$, which is generally used as a physical quantity of the tisue stiffness, and the shear modulus $G$ have the following relationship.

5 $$E=2\left(1+\nu \right)G,$$

where $\nu$ is the Poisson’s ratio. Since soft tissue is close to an incompressible medium, in which case the Poisson's ratio is close to 0.5, the relationship between Young’s modulus and the shear modulus in soft tissue is expressed by Eq. (6).

6 $$E\cong 3\rho {{c}_{s}}^{2}.$$

Assuming that the density of soft tissue is close to that of water and is constant at approximately 1000 kg/m^3^, Young’s modulus (unit: kPa) can be converted from the SWS ${c}_{s}$ obtained by Eq. (3). In the case of MRE, the elastic modulus distribution may be reconstructed from the displacement distribution of the shear wave by an inverse problem method [57]. Although the reconstruction in MRE is often a complicated problem, in the case of ultrasound SWE, the calculation of the elastic modulus is relatively simple.

## Factors potentially affecting SWE measurements

In interpreting SWE measurements, it is important to understand the factors that potentially affect the measurements. There is a report of a study that investigated the effects of gender, age, body mass index (BMI), measurement depth, ROI size, and other factors on measurement results [58]. On the other hand, the physics that affect shear wave propagation are also important [59]. As an example, the interrelationships of SWS, attenuation, and SWS dispersion in the viscoelastic body have been investigated on a model basis [60, 61]. Comprehensively, the factors that potentially affect SWE measurements may be categorized as physical and engineering factors. Physical factors include the propagation properties of shear waves, the mechanical properties of tissues [62], and the size and shape of target tissues. Engineering factors include the settings of the ultrasound system that generates and detects shear waves. Typical factors with respect to physics and engineering are described below.

### Physical factors

#### Propagation properties of shear waves

Shear waves are a type of wave. Therefore, shear waves refract in accordance with Snell's law. That is, when a shear wave is obliquely incident to the boundary between media having different SWSs, the propagation direction of the shear wave changes. In addition, interference, diffraction, and attenuation appear, as in the case of longitudinal waves. These may be the cause of artifacts in SWE. Also, a shear wave cannot propagate in perfect liquids.

Calculation of SWS in an ROI is often performed under the assumption that the shear waves propagate in the direction perpendicular to the progressive direction of the push pulse. However, the actual propagation of shear waves in the ROI does not always propagate in the perfectly lateral direction because of the above-mentioned propagation properties of shear waves. Therefore, it is conceivable that the measured value of SWS may be overestimated or underestimated in some cases.

#### Mechanical properties of tissues

Since the SWS is directly related to the shear modulus, the mechanical properties of the tissue (biomechanics) may be important factors for the interpretation of SWS. The SWS is converted to the Young’s modulus based on the assumption that the soft tissue is an isotropic linear elastic body. On the other hand, typical properties that break this assumption are (a) viscoelasticity, (b) nonlinearity, and (c) anisotropy. These have been reported to affect the SWS measurement, but attempts have also been made to utilize their properties for diagnosis.

**Fig. 4:**
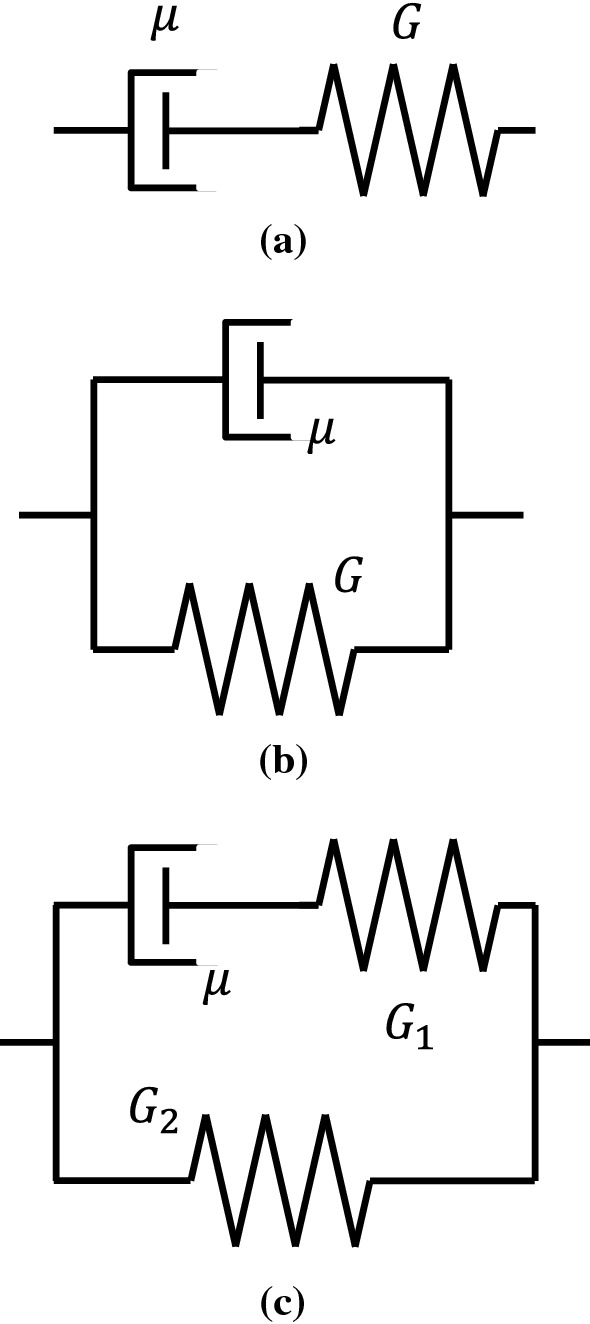
Typical viscoelastic models [62]. **a** Maxwell model, **b** Voigt model, **c** Kelvin model. Here, $G$, ${G}_{1}$, and ${G}_{2}$ indicate the shear modulus, and $\mu$ indicates the viscosity coefficient

**Fig. 5:**
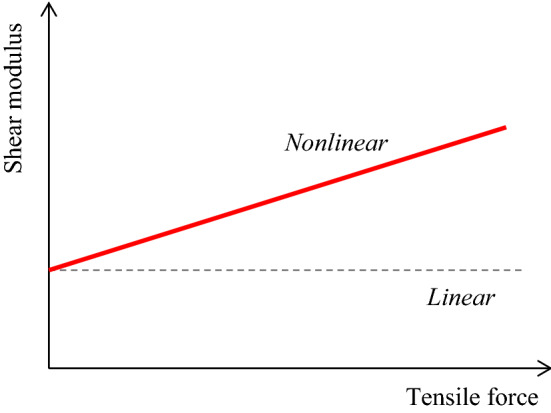
Nonlinear property of the measured SWS when load was applied along the direction of the muscle fibers. (Conceptual diagram of [75])
  Isotropy is a property where the elastic modulus does not change depending on the direction. However, tissues generally show anisotropy where the elastic modulus changes depending on the direction, like muscle fibers [62]. Results suggesting that the SWS reflects the anisotropy shown in Fig. 6 have been reported [77–81]. Gennisson et al. suggested the existence of anisotropy where SWSs differed depending on the direction of shear wave propagation to the renal cortex and medulla [77]. According to verifications by theoretical simulations and experiments in muscles, it has been reported that there are slow and fast SWSs in the biceps, and anisotropy where the SWS differs depending on the direction of muscle fibers has been reported [78, 79]. There are some attempts to use anisotropy for diagnosis. In echocardiography-based SWE, using the property where the SWS differs depending on the myocardial fiber orientation in pigs and sheep, i.e., anisotropy where the shear wave propagates faster along the fiber than across the fiber, the fiber orientation was mapped [80]. Similarly, anisotropy in rat brain has been mapped [81].

- *Viscoelasticity*
  Originally, elasticity is an ideal concept and does not include the time term in the relationship between the applied force and resultant deformation. However, actual tissue does not suddenly reach the final displacement state but shows a transient response that gradually reaches the final displacement state over time, so it is regarded as a viscoelastic body [62]. The phase of the periodic force differs from the phase of response displacement, and the resultant phase shift depends on the frequency. During propagation of shear waves in a viscoelastic body, since the phase velocity differs depending on the frequency, the elastic modulus converted from the measured SWS is affected by the phase velocity for each frequency of the shear wave. In general, the higher the frequency of the shear wave, the higher the elastic modulus value. More strictly, this elastic modulus is referred to as the complex modulus influenced by viscosity [57].
  Viscoelasticity is expected to be a useful diagnostic property rather than a property causing artifacts [63–66]. Other than SWE applications, there were attempts in the past to evaluate viscoelasticity based on the diffusion coefficient [67] measured by magnetic resonance imaging (MRI) and hysteresis property [68]. However, when using shear waves, velocity dispersion where the phase velocity differs depending on the frequency of the shear waves is utilized. The relationship between the frequency and the phase velocity of shear waves is referred to as a dispersion curve and is obtained by calculation based on the two-dimensional Fourier transform [69, 70]. As shown in Fig. 3, the slope of the dispersion curve is affected by viscosity. A flat line for no viscosity, a gentle slope for low viscosity, and a steep slope for high viscosity are observed. Further, the elastic and viscosity coefficients can be determined by the curve fitting method using typical viscoelasticity models, as illustrated in Fig. 4. In a study with another perspective, it was predicted that the dispersion slope in the range of a specific frequency would reflect the tissue microstructure, and it suggests that the dispersion slope can be useful for diagnosis [71].
- *Nonlinearity*
  Linearity is a property in which the resultant deformation is proportional to the applied force. That is, under slight deformation, the elastic modulus, which corresponds to the slope of the force–deformation relationship, becomes constant. However, tissues generally show nonlinearity in which force and deformation are not proportional [62]. Note that when the relationship between the elastic modulus and deformation (equivalent to strain) exhibits an exponential curve, the elastic modulus is proportional to the applied force (equivalent to stress). Consequently, the elastic modulus increases as the strain or stress increases. Other than SWE applications, there were attempts in the past to evaluate nonlinearity in strain imaging under static compression [72].
  Similarly, even when using shear waves, it has been reported that the SWS varies depending on the magnitude of strain or stress generated before SWS measurements (that is, initial state) [73–76]. There was a high correlation between the Young's modulus-strain relationship obtained using a tensile testing machine and the shear modulus-strain relationship obtained using SWE, and the shear modulus increased exponentially depending on the magnitude of strain [73]. In addition, as a result of applying a tensile load (passive muscle force) in the direction of muscle fibers and measuring the SWS, the SWS and corresponding shear modulus increased proportionally as the tensile force increased, as shown in Fig. 5 [74, 75]. This result is consistent with the above-mentioned relationship where the elastic modulus is proportional to the applied force. Therefore, the SWS may reflect the nonlinearity in the force–deformation relationship. Gennisson et al. theoretically and experimentally demonstrated that the SWS reflected nonlinearity [76].
- *Anisotropy*
  **Fig. 3:**
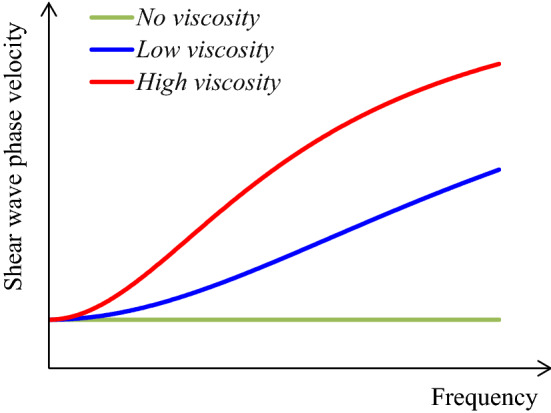
An illustration of a dispersion curve showing the velocity dispersion in which the phase velocity of shear waves differs depending on the frequency of the shear wave propagating in the viscoelastic body. A flat line for no viscosity, a gentle slope for low viscosity, and a steep slope for high viscosity are observed. (Conceptual diagram of [71])

**Fig. 6:**
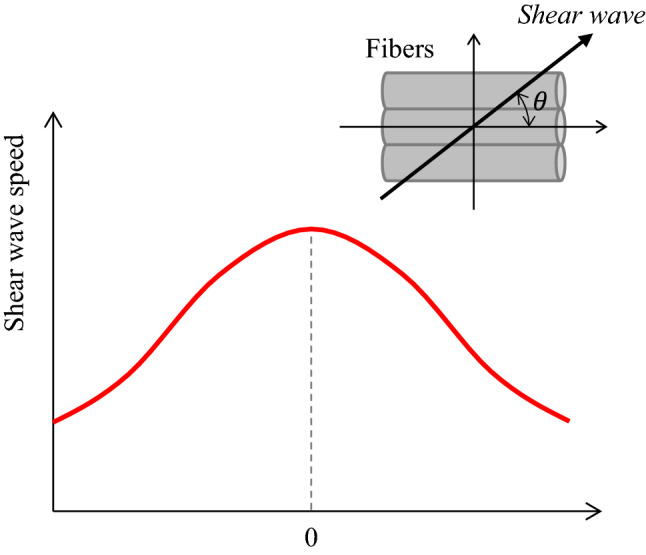
Anisotropy in which the SWS differs depending on the angle θ formed by the fiber orientation and the direction of shear wave propagation. (Conceptual diagram of [80])

#### Size of target tissue

Since the detectable size of the target tissue depends on the spatial resolution in shear wave imaging [82, 83], it may correspond to an engineering factor described below. However, due to the effect of the above-mentioned propagation properties of shear waves (e.g., internal reflection, refraction, interference, attenuation), the SWS may be affected by the size of the target tissue. Therefore, the effect of the size of target tissues such as tumors on the SWS is regarded as a physical factor in this review paper. In particular, the effect of target tissue whose size is smaller than the spatial resolution may be difficult to remove by signal processing in the ultrasound device. Ito et al. performed a two-dimensional shear wave propagation simulation based on a model simulating the microstructure of fibrous tissue and fat droplets. Their results suggested that wavefront discontinuity of shear waves may be related to the presence of minute fat droplets and affect the SWS [84].

#### Shape of target tissue

In a homogeneous medium, the shear wave generated from one focal point spreads like a spherical wave. However, it is expected that the propagation mode of the shear wave will be different at the interface between media with different acoustic impedances. Moreover, the SWS may be affected by the shape of the target tissue. So far, in particular, the effect of thin-layered media on the SWS has been investigated. Jang et al. reported that the SWS could not be measured correctly by the conventional TOF method because the shear wave propagates as a guide wave in the thin-layered media [85]. Similarly, Sadeghi et al. reported that the thickness exerted an unignorable effect on the SWS measurement, and the SWS decreased in thin-layered tissues such as fascia and aponeurosis [86]. They also proposed a correction method to obtain the correct SWS [85, 86].

### Engineering factors

As mentioned previously, in the typical SWS measurement procedure and elastic modulus calculation, first, the position of the focal point is determined near the ROI and a push pulse is transmitted to generate the shear wave centered on the focal point. Next, at different positions in the lateral direction in the ROI, the particle displacement is measured in the axial direction of the ultrasonic beam to reproduce the shear wave propagation. Only the progressive shear wave is extracted by the directional filter, and then the SWS is determined by the TOF method. Additionally, the elastic modulus is calculated. In general, the magnitude of the shear wave is influenced by the output intensity of the push pulse and affects the detectability of the shear wave. In addition, in shear wave detection processing, it is considered to be affected by factors such as the characteristics of the directional filter and the size of the kernel in the tracking algorithm. However, it may be difficult to specify the measurement algorithm of the SWS in commercially available equipment, and there is an attempt to calibrate the SWS by evaluating the difference between the equipment [87]. Others are equipped with a function to evaluate the reliability of the measured SWS [88]. Relatedly, a pixel-based quality evaluation method for 2D SWE has also been proposed separately [89]. Here, some estimable system-side settings (engineering factors) that affect the measurement of SWE are described.

#### Depth of ROI

Kaminuma et al. reported that the measurement accuracy of the SWS depends on the kind of probe used and the depth of the ROI (measurement depth), and that it is necessary to select the probe according to the depth of each lesion [90]. Zhao et al. also showed that the SWS measured using the TOF method could depend on the kind of probe (linear or convex), depth, and lateral tracking range [91]. As a reference, it has been reported that the reproducibility of ARFI imaging also changes depending on the measurement depth, and that measurement at a depth of 3–5 cm is suggested [92]. In addition, according to the results of a survey on the effect of probe frequency and measurement depth, to suppress SWS measurement fluctuations, it was suggested that high-frequency probes (linear) be used for a depth of 2–3 cm, and that low-frequency probes (convex) be employed for a depth of 4–5 cm [93]. These tendencies are also supported by another report [94]. However, these suggested depths may vary depending on the frequency of the push pulse. In other words, these results may suggest that SWS measurement accuracy is affected by the focal depth set on the device. As a possible mechanism, it is predicted that, since the in situ intensity of the push pulse is affected by the attenuation depending on the ROI depth, the in situ intensity decreases at a deeper focal point and the detection sensitivity for the resultant smaller shear wave decreases.

To improve the detection sensitivity, it may be effective to increase the output intensity of the push pulse. Deng et al. evaluated the relationship between the push pulse energy with two mechanical indexes (1.6 and 2.2; although 2.2 exceeds the regulation limit, note that this is a research setting) and the success rate of SWS measurement in the liver [95]. They found that the success rate of SWS measurement improved in proportion to the push pulse energy. That is, SWS measurements using a higher-energy push pulse were successful in patients in whom in SWS measurement failed using a push pulse with standard energy. Apart from the safety discussion, this result suggests that the output intensity of the push pulse may affect SWS measurement accuracy.

#### Signal processing

Generally speaking, since the amplitude of shear waves is small and susceptible to electrical noise, SWS measurement accuracy inevitably depends on the signal processing method (measurement algorithm). Rouze et al. reported that the size of the kernel used to measure the SWS and the method of determining the arrival time of the shear wave affect SWS measurement accuracy in 2D SWE [96]. Deng et al. investigated system-dependent factors that could affect SWS measurement through simulations and experiments. They found that SWS measurement errors could be suppressed to less than 3% in a system in which the processing for detecting the shear wave was properly adjusted [97].

## Verification method

Simulations and phantom experiments are effective for investigating factors that affect the SWS and verifying the hypotheses with respect to the interpretation of the measured SWS and the mechanism of shear wave propagation. In a simulation study, a simulator of shear wave propagation using the finite element method has been studied [98, 99], which is helpful for understanding the three-dimensional propagation of shear waves in a medium model.

Recently, using a research platform in which the SWE sequences were incorporated, an environment in which experimental verification using phantoms and so on can be easily performed was built [100]. At the same time, phantoms suitable for evaluating the performance of SWE are also important. For example, Nguyen et al. fabricated a viscoelasticity phantom mixing castor oil with gelatin [101]. While graphite powder is often mixed into the phantom material as scatterers for shear wave tracking, it has been reported that increasing the graphite concentration increases the SWS [102]. As a reference, a polyacrylamide gel-based phantom has also been fabricated [103]. On the other hand, there is no gold standard method for verifying the correctness of the SWS in viscoelasticity evaluation. Therefore, shear wave dispersion ultrasound vibrometry (SDUV) [104, 105] may be a potential gold standard for validation of viscoelasticity measurements in phantoms.

## Studies on the safety of SWE

Finally, studies on the safety of SWE are discussed. As mentioned previously, because the duration of the push pulse is longer than that of the conventional image pulse, the temperature rise related to biological effects has been investigated [106–110]. According to these studies, push pulse transmission to soft tissue does not fundamentally seem to cause significant temperature rises. On the other hand, it has been reported that the temperature rise becomes greater than that in the soft tissue when the focal point of the push pulse is on the bone surface, according to simulations and experiments using extracted animal bone [106, 109, 110]. In addition, it has been reported that non-serious arrhythmia tends to occur when ultrasound contrast agents and push pulse irradiation to rabbit heart are used in combination [111–113]. Further, it has also been reported that bleeding may occur when the push pulse is irradiated on the lung surface of rabbits [114].

## Conclusions

In this review paper, physical and engineering factors that potentially affect the SWS and elastic modulus were investigated based on SWE principles and a literature survey, with the aim of providing information for readers to formulate and verify the hypothesis with respect to the interpretation of SWE results. In the future, it is expected that technical understanding of SWE will deepen, and that SWE will become an indispensable diagnostic method in the clinical setting.
