# Correction to: A review of physical and engineering factors potentially affecting shear wave elastography

**DOI:** 10.1007/s10396-021-01159-2

**Published:** 2021-10-20

**Authors:** Naotaka Nitta, Makoto Yamakawa, Hiroyuki Hachiya, Tsuyoshi Shiina

**Affiliations:** 1 Health and Medical Research Institute, National Institute of Advanced Industrial Science and Technology (AIST), 1-2-1 Namiki, Tsukuba, Ibaraki 305-8564 Japan; 2 Graduate School of Medicine, Kyoto University, Kyoto, 606-8507 Japan; 3 School of Engineering, Tokyo Institute of Technology, Meguro, Tokyo, 152-8552 Japan

## Correction to: Journal of Medical Ultrasonics 10.1007/s10396-021-01127-w

The article A review of physical and engineering factors potentially affecting shear wave elastography, written by Naotaka Nitta, Makoto Yamakawa, Hiroyuki Hachiya and Tsuyoshi Shiina, was originally published electronically on the publisher’s internet portal on 28 August 2021 without open access. With the author(s)’ decision to opt for Open Choice the copyright of the article changed on 13 October 2021 to © The Author(s) 2021 and the article is forthwith distributed under a Creative Commons Attribution 4.0 International License, which permits use, sharing, adaptation, distribution and reproduction in any medium or format, as long as you give appropriate credit to the original author(s) and the source, provide a link to the Creative Commons licence, and indicate if changes were made. The images or other third party material in this article are included in the article’s Creative Commons licence, unless indicated otherwise in a credit line to the material. If material is not included in the article’s Creative Commons licence and your intended use is not permitted by statutory regulation or exceeds the permitted use, you will need to obtain permission directly from the copyright holder. To view a copy of this licence, visit http://creativecommons.org/licenses/by/4.0.

The original article has been corrected.

